# White-coat hypertension: management and adherence to guidelines by European and Canadian GPs. A cross-sectional clinical vignette study

**DOI:** 10.3399/bjgpopen19X101664

**Published:** 2019-10-02

**Authors:** Xavier Humbert, Sophie Fedrizzi, Emmanuel Touzé, Joachim Alexandre, Paolo-Emilio Puddu

**Affiliations:** 1 PhD Student, Department of General Medicine, Université Caen Normandie, Medical School, Caen, France; 2 GP, Pharmacovigilance Regional Center, CHU Caen, Caen, France; 3 GP, Pharmacology Department, CHU Caen, Caen, France; 4 GP, Medical School, Université Caen Normandie, Caen, France; 5 Pharmacist, Pharmacovigilance Regional Center, CHU Caen, Caen, France; 6 Pharmacist, Pharmacology Department, CHU Caen, Caen, France; 7 Medical School, Université Caen Normandie, Caen, France; 8 Physician and Professor, Medical School, Université Caen Normandie, Caen, France; 9 Physician and Lecturer, Pharmacovigilance Regional Center, CHU Caen, Caen, France; 10 Physician and Lecturer, Pharmacology Department, CHU Caen, Caen, France; 11 Physician and Lecturer, Medical School, Université Caen Normandie, Caen, France; 12 Physician and Professor, Department of Cardiovascular, Respiratory, Nephrological, Anesthesiological and Geriatric Sciences, Sapienza University of Rome, Rome, Italy; 13 Medical School, Université Caen Normandie, Caen, France

**Keywords:** white-coat effect, white coat hypertension, hypertension, blood pressure monitoring, ambulatory, guidelines, general practice, primary health care

## Abstract

**Background:**

White-coat hypertension (WCH) is also referred to as 'isolated clinic hypertension'. While it is a frequently encountered phenomenon, WCH is not systematically evoked, and its management remains unclear due to the contradictory guidelines provided by professional societies.

**Aim:**

To examine WCH management by GPs in Europe and Canada.

**Design & setting:**

A clinical vignette of a possible case of WCH was created from the literature, and the responses of GPs to WCH-specific questions in a cross-sectional electronic questionnaire were compared.

**Method:**

Complete electronic questionnaire responses from Europe and Canada were systematically analysed.

**Results:**

Among 770 eligible questionnaires (useful response rate: 10.6%), 43.5% were from France, 19.2% from Belgium, 7.8% from England, 19.5% from Switzerland, and 10.0% from Canada. Based on the clinical information provided in the vignette, GPs overall diagnosed hypertension and WCH equally (50.7% versus 49.3%, respectively). Canadian GPs suggested hypertension more frequently than European GPs in general (64.2% versus 46.1%, *P*<10^–4^), and more frequently used ambulatory blood pressure monitoring ([ABPM] 42.3% versus 26.1%, *P* = 0.01). In both groups of GPs, WCH was managed similarly (no treatment, 100% versus 97.3%, *P* = 0.39). Generally, the GPs all followed WCH patients for 3–6 months (51.3% versus 66.2%, *P* = 0.1), and they were not aware of the WCH guidelines (47.3% versus 52.1%, *P* = 0.54).

**Conclusion:**

Although WCH guidelines are different, WCH management by GPs is very similar except for diagnosis. Homogeneity in WCH guidelines is required and should be systematically implemented in hypertension guidelines to avoid inappropriate management of the condition.

## How this fits in

The consequences of WCH are controversial. Many contradictory guidelines have been published by professional societies, but the ‘real-life’ practices of GPs treating WCH are unknown. Despite these differences, this study shows that WCH is managed similarly in Europe and Canada.

## Introduction

Blood pressure (BP) status is traditionally based on office BP measurements. When both office (GP consultation) and out-of-office BP measurements (ABPM or home BP monitoring [HBPM]) are taken into account, four patient categories can be identified: true normotension (normal office and out-of-office BP), essential hypertension (both elevated office and out-of-office BP), masked hypertension (normal office BP and elevated out-of-office BP), and WCH.^1,2^

In 1984, Thomas Pickering wrote the original description of WCH.^3^ WCH, also called isolated office or isolated clinic hypertension, is used to describe patients who have increased clinic BP and normal BP outside the physician’s office.^4^

The overall prevalence of WCH in the general population is estimated to be approximately 10%–15%, and it amounts to 30% in patients with increased clinic BP recordings.^2^ WCH is more frequent in women, non-smokers, and patients with low clinic BP and smaller left ventricular mass at echocardiography.^5^ However, it is still controversial whether WCH represents a benign phenomenon. Some prospective studies exist regarding the association between WCH and cardiovascular disease (CVD) events, but the results are inconsistent. Over a median 10.6-year follow-up, Franklin *et al* ^6^ showed that subjects with low CVD risk and WCH have a CVD morbidity–mortality risk similar to that of age-adjusted normotensive control subjects (hazard ratio [HR] 1.06; 95% confidence intervals [CI] = 0.66 to 1.72, *P*=0.80). In contrast, Banegas *et al* ^7^ showed that WCH was associated with all-cause mortality (HR, 1.79; 95% CI = 1.38 to 2.32, *P*<0.001) in a multicentre national cohort that included 63 910 adults recruited from 2004–2014. Moreover, the results for CVD mortality were similar to those for all-cause mortality in the same study.^7^

Relatively old worldwide guidelines did not provide any specific recommendations regarding WCH management.^8–10^ More recently, the European Society of Cardiology, American Heart Association, and Hypertension Canada introduced a few guidelines for managing this phenomenon.^11–14^ The guidelines are unclear, however; there are some contradictions between the guidelines regarding WCH management, such as those produced in Canada^14^ compared to those in Europe.^12^ In this context, the authors examined WCH management by GPs as function of the country of practice: that is, France, Belgium, England, and Switzerland (grouped as 'Europe') and Canada.

## Method

### Electronic questionnaire

An electronic questionnaire was prepared in both French and English with three parts (Box 1). The first part referred to a clinical vignette of a typical case of essential hypertension and questions related to the case (diagnostic hypothesis, complementary exams, hypertension management). The case was a woman aged 60 years, without any smoking history, who has a BP of 170/90 at the beginning of the examination in the clinic. After a few minutes of rest, her BP becomes 150/90. She has a similar BP profile for 1 year. She is considered to be at low CVD risk because she has few cardiovascular risk factors (essential hypertension, female). The diagnosis of essential hypertension was given in the second part of the electronic questionnaire after a mean BP of 140/90 was obtained with HBPM. This second part of the questionnaire consisted of more specific questions related to WCH (diagnosis, characteristics, management) and presented the case of a patient with high CVD risk (past history of diabetes and hypercholesterolaemic disease). The last part of the electronic questionnaire contained questions related to basic demographic information: sex, year of birth, country (Belgium, Canada, England, France, or Switzerland), place of practice (urban, rural, or mixed urban–rural practice), type of practice (outpatient clinic, hospital, mixed [outpatient clinic and hospital], or replacement GP), and university activity (teacher and/or tutor). Excluded from the analysis were physicians with specific activity (full hospital activity or replacement GP), and questionnaires that were not completely filled out. Responders had the opportunity to formulate free comments at the end of the electronic questionnaire.

Box 1 **Electronic questionnaire**

**Clinical vignette**

A 60-year-old woman presents for a routine health review including a blood pressure (BP) check. She doesn’t smoke and takes no regular medication, she drinks occasional alcohol and says has little salt in her diet. On examination, the only abnormality found is that her BP is 170/90. A second BP taken after 5-minutes rest is 150/90.

From her medical record, her blood pressure 12 months ago was 150/90. She has no personal medical history. Nine months ago, she had a cholesterol of 5.8.

**Part 1: general questions**

Question 1: First, which diagnosis are you considering? (only one possible answer)

- Essential hypertension
- White-coat hypertension

Question 2: What do you want to do? (only one possible answer)

- Nothing
- Review at the practice
- Home monitoring
- Ambulatory monitoring

Question 3: If you had done home blood pressure monitoring (three measures morning, midday and evening, 3 days in a row) and the average blood pressure is 140/90, what is your conclusion? (only one possible answer)

- Normal blood pressure
- White-coat hypertension
- Essential hypertension
- Masked hypertension

**Part 2: specific questions**

Question 4: Finally, you prescribe for this patient ambulatory blood pressure monitoring. You observe normal blood pressure over 24 hours and your conclusion is white-coat effect. Do you treat this patient with an antihypertensive agent? (only one possible answer)

- Yes
- No

Question 5: What dietary advice do you give to this patient? (several possible responses)

- Reducing salt in diet
- Rationalise alcohol consumption
- Increasing of exercise

Question 6: If this patient was diabetic with a cholesterol of 6.2 and a family history of hypertension, would you treat this patient with an antihypertensive drug? (only one possible answer)

- Yes
- No

Question 7: How do you think this patient’s BP will progress over time?

- It will remain normotensive with a white-coat effect
- It will evolve towards essential hypertension

Question 8: How often would you plan to see this patient? (only one possible answer)

- Not routinely
- Within 3–6 months
- Within 1 year

Question 9: What would affect your decision to offer ambulatory BP monitoring to this patient? (several possible responses)

- Social security cover
- Patient's adherence to this management
- Difficulty of therapeutic education (patient would not take advice on having her BP managed by eg, medication)

Question 10: What would affect your decision to offer home BP monitoring? (several possible responses)

- Social security cover
- Patient's adherence to this management
- Difficulty of therapeutic education (patient would not take advice on having her BP managed by eg, medication)

Question 11: Are you are of any national guidance on the management of white-coat hypertension? (only one possible answer)

- Yes, I am aware of and use the guidance
- I know there are guidelines but I don’t routinely use them
- I do not think there is any guidance on the management white-coat hypertension
- I know there is no guidance on the management of white-coat hypertension

**Demographic questions**

Question 12: Would you describe yourself as (only one possible answer):

- Female
- Male
- Other/prefer not to answer

Question 13: What is your year of birth? (only one possible answer)

[dropdown menu , options from 1940 to 1990]

Question 14: In which country are you practising? (only one possible answer)

- Belgium
- Canada
- England
- France
- Switzerland

Question 15: How would you describe the area in which you work? (only one possible answer)

- Rural practice
- Urban practice
- Mixed urban and rural practice

Question 16: What is your mode of practice? (only one possible answer)

- Outpatient clinic
- Hospital
- Mixed (outpatient clinic + hospital)
- Replacement general practitioner

Question 17: Do you have a teaching and/or academic role? (only one possible answer)

- No, I do not teach, train, or hold an academic post
- I hold an academic post
- Yes, I am a trainer and offer training to doctors in my practice
- Yes, I am involved in undergraduate teaching in my practice
- Other:

### Mailing to GPs

After the validation of the electronic questionnaire by two independent French GPs and two independent French cardiologists trained in hypertension management, the form was sent by email to all GPs registered in national or regional databases (that is, *Cercles de médecins généralistes* in Belgium, the *Fédération des Médecins Omnipraticiens du Québec* in Canada, the NHS in England, the *Conseil départemental de l’Ordre des médecins* of 5 areas [Côtes d’Armor, Finistère, Morbihan, Orne, Seine-Maritime] in France, and the *Société Médicale de la Suisse Romande* in Switzerland), between 5 Oct 2017 and 27 Mar 2018. A reminder was sent 2 months after the initial message, and the study was closed on 27 Jun 2018. The participation of GPs was voluntary and anonymous. GPs were not paid to participate in this study. Substitutes for GPs or GPs without ambulatory activity were excluded during the analysis.

### Statistical analysis

Statistical analyses were performed using NCSS statistical software (version 12, 2018). Χ^2^ and student *t*-tests were used for the comparison of categorical and quantitative variables, respectively. The results were reported using beta, standard deviation (SD), and *P* values. A *P* value <0.05 was considered statistically significant.

## Results

### General results

From 5 Oct 2017 to 27 Mar 2018, *n* = 7263 questionnaires were sent out in Europe (France *n* = 4057; Switzerland *n* = 1303; Belgium *n* = 1151; and England *n* = 752) and *n* = 854 in Canada. In total, 973 questionnaires were returned (overall response rate: 12.0%), and 770 questionnaires (79.1%) were eligible for analysis (useful response rate: 10.6%). Of the returned questionnaires, 693 were from Europe (*n* = 335, 43.5% from France; *n* = 150, 19.5% from Switzerland; *n* = 148, 19.2% from Belgium; and *n* = 60, 7.8% from England), and 77 (10.0%) were from Canada. Overall, the mean GP age was 49.2 (SD 13.0) years, and 379 (49.2%) were male. Regarding other demographic data, GPs practised equally in rural and urban places (45.1% versus 44.9%, respectively), the main type of practice was in a group (73.2%), and 53.3% participated in university activities. Canadian GPs had the same sex distribution (*P* = 0.19) and the same mean age (*P* = 0.84) as European GPs. The place of practice of GPs in Canada and Europe was similar (*P* = 0.15), as was the participation in university activities (*P* = 0.37). Finally, Canadian GPs more frequently practised in an ambulatory group than European GPs (91.7% versus 72.5%, respectively; *P*<10^–4^).

### Clinical vignette

In the first part of the clinical vignette, regarding the diagnostic hypothesis, GPs suggested essential hypertension and WCH equally (50.7% versus 49.3%, respectively). In the second part (with a BP of 140/90 after HBPM), GPs mainly suggested essential hypertension (45.9%), followed by WCH (26.1%), normotension (18.9%), and masked hypertension (6.1%). Likewise, in the second part of the questionnaire, GPs used ABPM or HBPM for the WCH diagnosis (95.2%) and did not treat WCH (97.7%) in patients with a low CVD risk. In contrast, half of the GPs treated WCH in patients with a high CVD risk. They wished to follow the patients with WCH for 3–6 months (65.0%). Moreover, they mainly predicted an evolution towards essential hypertension (62.1%). Finally, the majority of GPs declared that no guidelines exist for WCH (52.2%).

### Comparisons between Canadian and European GPs (Figure 1)

In this specific analysis, Canadian and European GPs did not give the same diagnosis in the clinical vignette (part 1): Canadian GPs more frequently diagnosed essential hypertension than European GPs (64.2% versus 46.1%, respectively, *P*<10^–4^). They all used ABPM or HBPM to make the diagnosis (97.1% versus 95.3% for Canadian and European GPs, respectively, *P* = 0.76), but Canadian GPs used ABPM more frequently than European GPs (42.3% versus 26.1%, respectively, *P* = 0.01). In the second part, they used the same management approach to WCH (no treatment of WCH, 100% versus 97.3% for Canadian and European GPs, respectively, *P* = 0.39) even for a patient with high CVD risk (no treatment of WCH in patient with high CVD risk, 52.1% versus 51.3% for Canadian and European GPs, respectively, *P* = 0.9). Similarly, they followed WCH patients for 3–6 months (51.3% versus 66.2% for Canadian and European GPs, respectively, *P* = 0.1). They predicted the same evolution of WCH towards essential hypertension (61.4% versus 62.5% for Canadian and European GPs, respectively, *P* = 0.8). Finally, both Canadian and European GPs reported being unaware of WCH guidelines in their country ([Box 1, question 11, answer C] 47.3% versus 52.1%, respectively, *P* = 0.54).

## Discussion

### Summary

This study investigated the impact of WCH guidelines on primary care in Europe and Canada. Canadian GPs more frequently diagnosed essential hypertension, but they adopted an attitude towards WCH management similar to that of the European GPs, even for a patient with high CVD risk. All GPs followed up similarly with WCH patients for 3–6 months.

### Strengths and limitations

This study is the first to investigate what the impact of the WCH guidelines has been on the daily practice of GPs internationally. Few studies have previously investigated the practices of physicians regarding the diagnosis and management of hypertension, and its distinction from WCH. In 2000, Hyman and Pavlik^15^ showed that 43% of physicians had higher BP thresholds for the diagnosis and treatment of hypertension than the 140/90 mmHg criterion.^15^ Although recommendations regarding the management of WCH exist, they are only available in a few sets of hypertension guidelines,^4,11,12,14^ and they are completely absent in others.^8–10^ Moreover, whereas WCH guidelines have been published for several years, they are often not known to physicians. Indeed, half of GPs in the present study were unaware of their existence.

The main weakness of this study is the risk of inadequate representativeness due to the low participation rate (10.6%), particularly in Canada. This is a commonly occurring problem of primary care surveys,^16^ particularly with unpaid studies. However, several reviews have noted that low response rates in GP surveys do not necessarily introduce selection bias.^17,18^ Moreover, a clinical vignette is a validated tool for assessing variation in physicians’ practices.^19^ The authors could not conduct the same study in other places of interest where WCH guidelines exist (the US or Japan, for example). Only GPs were studied, but similar WCH diagnostic and management difficulties may be encountered by other physicians, such as cardiologists or nephrologists. Nevertheless, good theoretical knowledge on WCH does not seem to prevent misdiagnosis: a better understanding of WCH guidelines is, therefore, much needed.

### Comparisons with existing literature

The management of WCH is different in the Canadian and European guidelines. In randomised controlled trials (RCTs) of BP follow-up strategies, patients in whom antihypertensive medications have either been reduced or stopped are thought to represent individuals with WCH. Staessen *et al* ^20,21^ have already shown in two RCTs that the use of HBPM to diagnose WCH permits a significant reduction in antihypertensive medication use without changes in other clinical cardiovascular surrogate outcomes (that is, electrocardiographic and echocardiographic left ventricular mass and cardiovascular symptoms) compared to use of office BP measurements only (25.6% versus 11.3%, respectively; *P*<0.001), but only in short follow-up studies of 182–365 days. Consequently, according to Canadian guidelines, if the out-of-office average BP is not elevated, WCH should be diagnosed and pharmacologic treatment should not be initiated^14^ because WCH is considered to be without any CVD-related consequences. Additionally, antihypertensive drugs have been shown to effectively and persistently lower office BP, with no concomitant reduction of ambulatory BP values.^22,23^ However, whether these BP changes lead to CVD protection has not been investigated by adequately powered studies, and thus the answer to this question remains unknown. It should be considered inevitable that people with WCH have been represented in RCTs documenting the protective effect of antihypertensive drugs.^12^ In a recent subanalysis of the HYVET trial, Bulpitt *et al* ^24^ showed that in the oldest hypertensive patients (aged over 80 years), WCH was present in 55% of the trial population. Consequently, antihypertensive drug treatment cannot definitively be excluded for patients with WCH because few studies have been performed investigating the effect of antihypertensive drug treatment specifically on this patient group, including studies with long-term follow-up. Mancia *et al* ^25^ suggest that WCH is not benign and might warrant medication. During a 10-year follow-up, 42.6% of patients with WCH developed hypertension. Compared with normotensive subjects, adjusting for age and sex, the risk of becoming hypertensive was significantly higher for WCH subjects (odds ratio 2.51; *P*<0.0001).^25^ Overtreatment of patients with WCH with antihypertensive drugs has a medico-economic impact, while effectiveness is uncertain. European guidelines explain that an antihypertensive treatment may be considered in patients with WCH, particularly for people with evidence of hypertension-mediated organ damage, or with a high or very high CVD risk.

BP varies during the day and is also different between the clinic and home, even in normotensive patients. An out-of-office BP measurement refers to a measurement obtained with the use of either HBPM, or ABPM. This type of monitoring provides a larger number of BP measurements compared to conventional office BPs in conditions that are more representative of daily life.^26^ The real difficulties are the different definitions of hypertension and whether it is defined according to office BP, ABPM or HBPM. Hypertension is definite when office BP ≥140/90 mmHg, or from HBPM ≥135/85, or the 24-hour mean BP from ABPM ≥130/80, or the night-time mean BP from ABPM ≥120/70, or the daytime mean BP from ABPM ≥135/85. These different cut-offs are confusing in daily practice.^12^ Misdiagnosis might lead to inappropriate prescriptions and, consequently, extra costs (in medications and office visits), especially in cases of side effects from medications in older patients with multiple comorbidities.^26^ The European Society of Hypertension has also described the classification of office BP measurements and definitions of hypertension grades from grade one hypertension (systolic blood pressure [SBP]: 140–159 and/or diastolic blood pressure [DBP]: 90–99), to grade two hypertension (SBP: 160–179 and/or DBP: 100–109), to grade three hypertension (SBP ≥180 and/or DBP ≥110).^12^

Regarding follow-up with WCH patients, Canadian guidelines indicate that physicians should plan an annual follow-up visit. In contrast, in Europe, adapted follow-up for WCH patients is less frequent (no less than every 2 years). Despite these guidelines, it is interesting to note that GPs mostly followed-up with WCH patients every 3–6 months (51.3% versus 66.2% for Canadian and European GPs, respectively, *P* = 0.1) in the present study. Indeed, 42.6% of patients with WCH will develop essential hypertension within a few years.^11,25^ Consequently, evolution towards high blood pressure can be more precisely detected with this frequent follow-up schedule.

### Implications for research and practice

As GPs are frequently confronted with WCH, they should be aware of this phenomenon. Guidelines for the treatment of WCH must be better known, particularly regarding the use of ABPM or HBPM to improve the diagnosis. Finally, attitudes concerning the treatment of WCH remain ambiguous. Well-designed studies, such as RCTs, must be performed in primary care settings to clarify WCH management, as was done in the HYVET2 study, for example.^27^ Moreover, a comparison with secondary care settings may also be interesting.

In conclusion, guidelines for WCH management and follow-up are different in Canada and Europe. However, management of WCH by GPs is very similar, except for diagnosis. Homogeneity in WCH guidelines is definitely required and should be systematically implemented in hypertension guidelines to avoid potential inappropriate management of the condition.

**Figure 1.**
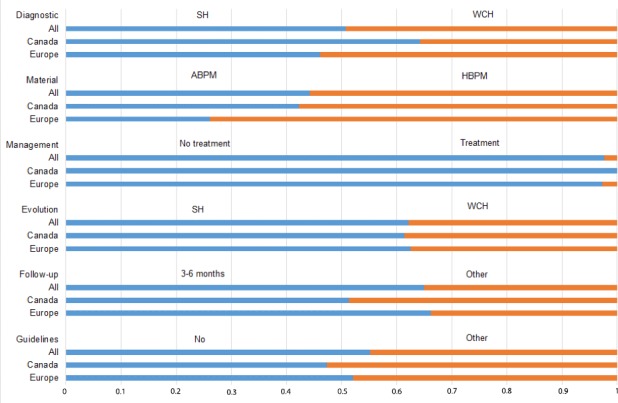
Comparisons between Canadian and European GPs concerning clinical vignette responses ABPM = ambulatory blood pressure monitoring. HBPM = home blood pressure monitoring. SH = sustained (essential) hypertension. WCH = white-coat hypertension.
